# Predicting personality disorder functioning styles by the Chinese Adjective Descriptors of Personality: a preliminary trial in healthy people and personality disorder patients

**DOI:** 10.1186/s12888-016-1017-0

**Published:** 2016-08-30

**Authors:** Hongying Fan, Qisha Zhu, Guorong Ma, Chanchan Shen, Bingren Zhang, Wei Wang

**Affiliations:** 1 Department of Clinical Psychology and Psychiatry/ School of Public Health, Zhejiang University College of Medicine, Yuhangtang Road 866, Hangzhou, Zhejiang 310058 China; 2 Department of Psychology and Behavioral Sciences, Zhejiang University College of Sciences, Hangzhou, China

**Keywords:** Chinese culture, Personality disorder patients, The Chinese Adjective Descriptors of Personality (CADP), The Parker Personality Measure (PERM)

## Abstract

**Background:**

Cultural and personality factors might contribute to the clinical differences of psychiatric patients all over the world including China. One cultural oriented Chinese Adjective Descriptors of Personality (CADP) designed to measure normal personality traits, might be specifically associated with different personality disorder functioning styles.

**Methods:**

We therefore have invited 201 healthy volunteers and 67 personality disorder patients to undergo CADP, the Parker Personality Measure (PERM), and the Plutchik-van Praag Depression Inventory (PVP) tests.

**Results:**

Patients scored significantly higher on PVP scale and all 11 PERM personality disorder functioning styles, as well as CADP Emotional and Unsocial traits. The PVP was significantly correlated with some CADP traits and PERM styles in both groups. In healthy volunteers, only one CADP trait, Unsocial, prominently predicted 11 PERM styles. By contrast in patients, CADP Intelligent predicted the PERM Narcissistic and Passive-Aggressive styles; CADP Emotional the PERM Paranoid, Borderline, and Histrionic styles; CADP Conscientious the PERM Obsessive-Compulsive style; CADP Unsocial the PERM Schizotypal, Antisocial, Narcissistic, Avoidant, Dependent, and Passive-Aggressive styles; CADP Agreeable the PERM Antisocial style.

**Conclusion:**

As a preliminary study, our results demonstrated that, in personality disorder patients, all five CADP traits were specifically associated with almost all 11 personality disorder functioning styles, indicating that CADP might be used as an aid to diagnose personality disorders in China.

## Background

There is evidence that the psychiatric disorder patients display some clinical or epidemiological differences all over the world including China. For instance, Chinese adolescents have experienced more depression than Japanese and Korean adolescents did [1], Chinese elders have the lowest standardized prevalence of sleep complaints amongst six developing countries such as Cuba, Mexico, and India [2], Chinese patients with major depressive disorder have lower suicidality tendency than those in South Korea [3], and Chinese bipolar disorder patients have a remarkably lower comorbidity of alcohol problems than the Western bipolar patients did [4]. Moreover, Huang Y et al. [5] found that the prevalence of personality disorders in China were higher than that in Western Europe but lower than that in USA.

One might easily suspect that all the above-mentioned differences are associated either with the uniqueness in Chinese culture, or with the personality trait differences. Recently, a questionnaire based on the Chinese adjective pool, the Chinese Adjective Descriptors of Personality (CADP) [6], has been developed to measure the normal traits of Intelligent, Emotional, Conscientious, Unsocial and Agreeable, which are comparable to the traits measured by the five-factor model of personality, the Openness to Experience, Neuroticism, Conscientiousness, Extraversion, and Agreeableness respectively. Since its development is based on the Chinese culture, CADP might be more suitable to measure the personality-related psychiatric disorders in China. Recently, it has been demonstrated that the CADP traits were correlated respectively with the clinical symptoms of two types of bipolar disorder in Chinese culture [7].

Empirical evidence has established the relationships between normal and disordered personality traits, for instance, between the normal personality traits measured through the five-factor model such as the NEO-PI-R [8] and the clinically defined personality disorders in the general and clinical populations [9, 10]. A recent study [11] has also shown that the alternative five-factor model of normal traits measured by the Zuckerman-Kuhlman Personality Questionnaire (ZKPQ) [12] were associated with the personality disorder functioning styles measured by the Parker Personality Measure (PERM) [13]. The question therefore arises whether the CADP normal traits are associated with the disordered personality in Chinese healthy volunteers as well as in personality disorder patients.

It has been suggested that the abnormal personalities are the extremes of normal personality variation [14, 15]. Numerous results indicate that personality-descriptive models such as the five-factor model are able to capture substantial variance of personality pathology in adults [16] and in adolescents [17, 18]. For instance, Openness to Experience is significantly correlated with personality disorders such as the paranoid, schizotypal [19], obsessive-compulsive, narcissistic, and antisocial personality disorders [20]. Neuroticism is correlated with the paranoid, schizotypal, antisocial, borderline, narcissistic, and obsessive-compulsive personality disorders [20]. Conscientiousness is associated with the borderline, antisocial, obsessive-compulsive [20], and passive-aggressive [21] personality disorders. Extraversion is highly consistent with the schizotypal, narcissistic, antisocial, avoidant, and passive-aggressive personality disorders [22, 23]. Agreeableness is associated with the antisocial, narcissistic, and dependent personality disorders [8, 22]. We thus hypothesized that (1) CADP Intelligent would be correlated with the PERM Paranoid, Schizotypal, Antisocial, Narcissistic, and Obsessive-Compulsive personality disorder functioning styles; (2) Emotional, the Paranoid, Schizotypal, Antisocial, Borderline, Narcissistic, and Obsessive-Compulsive styles; (3) Conscientious, the Borderline, Antisocial, Obsessive-Compulsive and Passive-Aggressive style; (4) Unsocial, the Schizotypal, Narcissistic, Antisocial, Avoidant, and Passive-Aggressive styles; (5) Agreeable, the Antisocial, Narcissistic, and Dependent styles; and (6) these correlations would be more pronounced in personality disorder patients. We therefore have invited both healthy volunteers and personality disorder patients to undergo tests of CADP and PERM in the current study.

## Methods

### Participants

Two hundred and one healthy participants (113 women, 88 men; mean age, 20.56 years ± 1.31 S.D., age range, 18-26 years) and 67 patients with personality disorders (38 women and 29 men; mean age, 20.78 ± 1.25, age range, 18-24). The healthy participants were determined not suffering or had suffered from any neurological or psychiatric problems through a semi-structured interview. The semi-structured interview was developed by the authors, which covered major classifications of neurological and psychiatric disorders, streamed with possible major complaints from clinical patients. Patients were firstly diagnosed through the DSM-IV-TR criteria [24] by an experienced psychiatrist (WW). Once a patient was diagnosed as suffering from a personality disorder, s/he would be handed over to other two co-authors (HF and CS) for a later confirmation using the Structured Clinical Interview for DSM-IV Axis II (SCID-II). The DSM-IV-TR diagnostic procedure was served as a participant-selection filter, and the SCID-II procedure was served as a diagnostic accuracy verification for the study design. Overall, over 90 % congruency was reached between the DSM-IV-TR and SCID-II diagnostic labels to the recruited patients. Patient distributions were: 3 patients with the paranoid, 15 schizoid, 3 schizotypal, 2 antisocial, 2 borderline, 13 histrionic, 2 narcissistic, 4 avoidant, 5 dependent, 6 obsessive-compulsive, 10 passive-aggressive, 1 comorbid schizoid and histrionic, and 1 comorbid paranoid, histrionic and avoidant personality disorders. Patients were free from organic brain lesions according to recent magnetic resonance imaging or computed tomography scans, and were free from any antipsychotic drugs or alcohol for at least 72 h before test. No age (*t* = 1.20, *p* = .85), gender (*χ*^*2*^ = .005, *p* = .94) or education level (*t* = .75, *p* = .46) differences were found between the two groups.

### Instruments

Participants were asked to complete the following questionnaires in a quiet room.

A. The Chinese Adjective Descriptors of Personality (CADP) [6] with 100 Chinese adjectives, is designed to measure the personality traits such as the Intelligent, Emotional, Conscientious, Unsocial and Agreeable (20 adjectives each). Participants were asked to complete the rating items using the Likert type scales: 1-very unlike me, 2-moderately unlike me, 3-somewhat like and unlike me, 4-moderately like me, and 5-very like me. The internal alphas of each CADP factor in the two groups were satisfactory (Table 1) as demonstrated previously [6, 7].

**Table 1:** Internal alphas and scale scores (Mean ± SD) of Chinese Adjective Descriptors of Personality (CADP) and the Parker Personality Measure (PERM)

	Healthy controls (*n* = 201)		Personality disorders (*n* = 67)		95 % Confidence Interval
	Alpha	Score	Alpha	Score	
CADP					
Intelligent	.96	63.91 ± 13.55	.96	60.12 ± 16.90	-7.81 ~ .224
Emotional	.92	51.11 ± 14.32	.92	61.93 ± 15.98*	6.46 ~ 15.17
Conscientious	.95	72.08 ± 13.50	.93	69.49 ± 14.46	-6.40 ~ 1.23
Unsocial	.90	36.79 ± 10.58	.88	48.72 ± 13.30*	8.38 ~ 15.48
Agreeable	.91	74.85 ± 11.29	.88	74.93 ± 11.44	-3.07 ~ 3.23
PERM					
Paranoid	.81	20.06 ± 6.14	.82	27.39 ± 7.87*	5.23 ~ 9.42
Schizoid	.53	19.63 ± 4.14	.59	22.48 ± 5.41*	1.42 ~ 4.29
Schizotypal	.67	9.46 ± 3.30	.66	13.69 ± 4.44*	3.06 ~ 5.40
Antisocial	.74	18.84 ± 5.28	.73	23.84 ± 6.56*	3.25 ~ 6.75
Borderline	.77	20.34 ± 5.91	.75	28.57 ± 7.51*	6.23 ~ 10.23
Histrionic	.61	11.61 ± 3.44	.58	16.16 ± 4.02*	3.47 ~ 5.64
Narcissistic	.78	15.53 ± 4.95	.63	20.30 ± 5.15*	3.35 ~ 6.20
Avoidant	.78	24.00 ± 6.15	.71	31.75 ± 6.84*	5.88 ~ 9.62
Dependent	.73	21.26 ± 5.57	.73	27.30 ± 7.19*	4.13 ~ 7.95
Obsessive-Compulsive	.68	16.07 ± 4.36	.61	20.00 ± 4.24*	2.73 ~ 5.12
Passive-Aggressive	.70	19.87 ± 5.20	.76	23.99 ± 6.79*	2.31 ~ 5.92

Note: * *p* < .05 vs. Healthy controls

B. The Parker Personality Measure (PERM) [13], which measures 11 functioning styles of paranoid, schizoid, schizotypal, antisocial, borderline, histrionic, narcissistic, avoidant, dependent, obsessive-compulsive, and passive-aggressive personality disorders. Each PERM item has the same 5-point Likert scale as used in CADP measure. The internal alphas of each PERM scale in the two samples were satisfactory (Table 1) as reported previously [11, 24].

C. The Plutchik-van Praag Depression Inventory (PVP) [25] contains 34 items; each item has a three-point scale (0, 1, 2), which corresponds to depressive tendencies. Participants have “possible depression” if they score between 20 and 25, or “depression” if they score higher than 25. The internal alpha of PVP in the present study was .89, which was also comparable to the previous report [25].

### Data analyses and statistics

Two-way ANOVA were applied to the five CADP trait or 11 PERM functioning style scores in the two groups (Group × Scale). Whenever a significant main effect was found, post-hoc analysis by the Student *t* test was employed to evaluate between-group differences. The Student *t* test was also employed to the mean PVP scores in the two groups. Inspired by a study [26], we applied the multiple linear regression analysis (stepwise method) to search for the relationships between the five CADP traits and the 11 PERM scales, taking CADP traits as potential predictors for the PERM scales. A p value less than .05 was considered to be significant. At the p level and with the present sample size (the smaller one, n = 67), power to detect a raw effect (such as a scale score difference) was 99.3 %, a column effect 73.4 %, and an interaction effect 99.3 %.

## Results

In both groups, the internal alphas of the five CADP traits and the 11 PERM functioning styles were satisfactory (Table 1). The mean PVP score in personality disorders (18.25 ± 10.97 S.D.) was higher than that in healthy controls (7.96 ± 5.55 S.D., *p* < .001, 95 % Confidence Interval (CI): 7.51 ~ 13.07).

Two-way ANOVA also has detected significant differences of the five CADP traits in the two groups (group effect, F [1, 266] = 11.87, *p* = .001; mean square effect (MSE) = 2717.17; scale effect, F [4, 1064] = 200.76, *p* < .001; MSE = 32253.45; and group × scale interaction effect, F [4, 1064] = 17.69, *p* < .001; MSE = 2841.60). The post-hoc Student *t* test has detected that personality disorders scored significantly higher than the controls did on the Emotional (*p* < .001) and Unsocial (*p* < .001) traits (Table 1).

Meanwhile, two-way ANOVA has detected that there were significant differences of the 11 PERM styles between the two groups (group effect, F [1, 266] = 105.22, *p* < .001; MSE = 15786.73; scale effect, F [10, 2660] = 287.45, *p* < .001; MSE = 4701.11; and group × scale interaction effect, F [10, 2660] = 9.39, *p* < .001; MSE = 153.58). The post-hoc Student *t* test has detected that personality disorders scored significantly higher than the controls did on the Paranoid, Schizoid, Schizotypal, Antisocial, Borderline, Histrionic, Narcissistic, Avoidant, Dependent, Obsessive-Compulsive, and Passive-Aggressive (all ps < .001) styles (also see Table 1).

In the healthy controls, PVP was significantly correlated with CADP Intelligent (*r* = -.22, *p* = .002) and Unsocial (*r* = .23, *p* = .001) traits, and with PERM Paranoid (*r* = .21, *p* = .003), Schizotypal (*r* = .17, *p* = .014), Borderline (*r* = .31, *p* < .001), Histrionic (*r* = .17, *p* = .017), Avoidant (*r* = .28, *p* < .001), Dependent (*r* = .28, *p* < .001), and Obsessive-Compulsive (*r* = .17, *p* = .014) functioning styles. In personality disorders, PVP was significantly correlated with CADP Intelligent (*r* = -.40, *p* = .001) trait, and with PERM Schizotypal (*r* = .35, *p* = .004), Borderline (*r* = .38, *p* = .002), and Avoidant (*r* = .29, *p* = .016) functioning styles.

Considering the prediction of PERM functioning styles by the CADP traits, the accounted variances (adjusted *R*^*2*^ values) were ranged from .19 to .49 in controls, and from .05 to .47 in personality disorders (Table 2). In personality disorders, all predictors of a given personality disorder functioning style were prominent (βs > .30): Intelligent predicted the Narcissistic and Passive-Aggressive styles; Emotional the Paranoid, Borderline, and Histrionic styles; Conscientious the Obsessive-Compulsive style; Unsocial the Schizotypal, Antisocial, Narcissistic, Avoidant, Dependent, and Passive-Aggressive styles; Agreeable the Antisocial style. In controls, Unsocial (the βs > .30) was the most prominent predictor of all the 11 personality disorder styles. For instance, Unsocial predicted the Paranoid (β = .53), Schizoid (.52), Schizotypal (.65), Antisocial (.45), Borderline (.54), Histrionic (.47), Narcissistic (.54), Avoidant (.60), Dependent (.54), Obsessive-Compulsive (.30) and Passive-Aggressive (.45) styles.

**Table 2:** Stepwise multiple regressions predicting the Parker Personality Measure styles with the Chinese Adjective Descriptors of Personality traits in the healthy controls and the personality disorders

	Healthy controls (*n* = 201)		Personality disorders (*n* = 67)	
	Adjusted *R* ^*2*^	β (*B*, *SE*), predictors	Adjusted *R* ^*2*^	β (*B*, *SE*), predictors
Paranoid	.34	.53 (.31, .04) Unsocial**	.47	.47 (.23, .05) Emotional**
		.14 (.06, .03) Emotional*		-.25 (-.17, .07) Agreeable*
				.21 (.12, .06) Unsocial*
Schizoid	.23	.52 (.20, .03) Unsocial**	.05	-.26 (-.09, .04) Emotional*
		-.15 (-.04, .02) Emotional*		
Schizotypal	.41	.65 (.20, .02) Unsocial**	.26	.38 (.13, .04) Unsocial**
				.27 (.07, .03) Emotional*
Antisocial	.49	.45 (.23, .03) Unsocial**	.39	.45 (.22, .05) Unsocial**
		-.28 (-.13, .03) Agreeable**		-.37 (-.21, .06) Agreeable**
		.33 (.13, .02) Intelligent**		.29 (.11, .04) Intelligent**
		-.25 (-.10, .03) Conscientious**		
		.16 (.06, .02) Emotional**		
Borderline	.43	.54 (.30, .03) Unsocial**	.26	.52 (.25, .05) Emotional**
		.17 (.07, .02) Emotional**		
		-.14 (-.07, .03) Intelligent*		
Histrionic	.32	.47 (.15, .02) Unsocial**	.18	.44 (.11, .03) Emotional**
		.21 (.05, .02) Emotional**		
Narcissistic	.38	.54 (.25, .03) Unsocial**	.14	.35 (.11, .04) Intelligent**
		.36 (.13, .02) Intelligent**		.34 (.13, .05) Unsocial**
		-.26 (-.12, .03) Agreeable**		
Avoidant	.36	.60 (.35, .03) Unsocial**	.17	.43 (.22, .06) Unsocial**
Dependent	.39	.54 (.29, .03) Unsocial**	.15	.36 (.22, .06) Unsocial**
		-.22 (-.09, .02) Conscientious**		
Obsessive-Compulsive	.19	.48 (.16, .03) Conscientious**	.24	.57 (.17, .04) Conscientious**
		.30 (.13, .03) Unsocial**		-.25 (-.06, .03) Intelligent*
		-.20 (-.08, .03) Agreeable*		
Passive-Aggressive	.36	.45 (.37, .06) Unsocial**	.21	.43 (.22, .06) Unsocial**
		-.25 (-.11, .04) Agreeable*		.36 (.15, .05) Intelligent**
		.24 (.09, .03) Intelligent*		-.25 (-.12, .06) Conscientious*
		-.22 (-.09, .03) Conscientious**		

Note: * *p* < .05, ** *p* < .01; βs valued larger than .30 were bolded for clarity; *B* and standardized error (*SE*) were unstandardized coefficients

## Discussion

This study explored the potential predictability of the CADP traits to the personality disorder functioning styles both in personality disorder patients and in healthy volunteers. The mean PVP score in personality disorders was higher than in healthy controls. Personality disorder patients scored significantly higher than the healthy volunteers did on the CADP Emotional and Unsocial traits and on all 11 PERM functioning styles. PVP was significantly correlated with CADP Intelligent (-) and Unsocial traits, and with PERM Paranoid, Borderline, Schizotypal, Histrionic, Avoidant, Dependent, and Obsessive-Compulsive functioning styles in healthy controls; while in personality disorders, it was significantly correlated with CADP Intelligent (-), and with PERM Schizotypal, Borderline, and Avoidant functioning styles. In personality disorder patients, all five CADP predictors of a personality disorder functioning style were prominent, which confirmed most of our hypotheses.

Patients scored significantly higher in PVP than the healthy volunteers did, which was in accordance with previous reports [27, 28]. The PVP was also correlated with PERM Avoidant in our patients, which was in line with the high co-morbidity between the avoidant personality disorder and the major depressive disorder in clinics [29–31]. In our healthy volunteers, PVP was correlated with Unsocial, which agreed with the notion that the Unsocial represents the negative poles of extraversion [32] or Social Life [33], and that extraversion was significant-negatively correlated with depression [34]. In our healthy participants, PVP was also correlated with PERM Paranoid and Obsessive-Compulsive functioning styles. Indeed, the major depressive disorder was frequently co-morbid with the paranoid and obsessive-compulsive personality disorders [35].

The Emotional and Unsocial were significantly elevated in our patients, which were in line with the well-established trends that some personality disorders such as the borderline, avoidant, and dependent types displayed higher neuroticism [36], and that some personality disorders such as the narcissistic and histrionic types had lower extraversion [9]. All PERM styles were elevated in our patients, which were in accordance with previous results in personality disorder patients [11].

The interesting results in our two groups also pointed to the predicting PERM styles with CADP traits. Firstly, the Paranoid, Borderline, and Histrionic styles were strongly associated with the Emotional in patients. As for other researches (e.g. using NEO-PI-R or ZKPQ), neuroticism was consistently high-correlated with the paranoid and borderline personality disorders [37, 38], and sometimes correlated with the histrionic personality disorder [39]. On the other hand, patients with paranoid, borderline, or histrionic personality disorder have less emotional understanding (i.e., emotional clarity), more difficulty to regulate their emotional states (i.e., emotional repair) and their emotional self-insights [40].

Secondly, the Schizotypal, Antisocial, Narcissistic, Avoidant, Dependent, and Passive-Aggressive personality disorder functioning styles were strongly predicted by the Unsocial trait in patients. Similarly, a relationship between the extraversion (negative pole of Unsocial) and the schizotypal (-), antisocial (-), narcissistic, avoidant (-), and dependent (-) personality disorders was reported in the healthy and patient samples [41, 42]. The direct explanation for the association between the Passive-Aggressive style and the Unsocial trait found in patients is still lacking, however, some evidence has shown that the avoidant [38] and passive-aggressive personality disorders were associated with neuroticism-anxiety [37]. Aluja et al. [38] found the correlations between most facets of extraversion and the paranoid (-) and obsessive-compulsive (-) types were rather weak, which worked in concert with the current results, except histrionic. In clinics, the histrionic personality disorder is characterized by an excessive emotionality and attention seeking [24], which contrasts with the Unsocial trait that is characterized by the stupid, dull or inflexible behavior [6].

Thirdly, the Narcissistic and Passive-Aggressive styles were strongly predicted by the Intelligent trait in patients. In one study with healthy students, the Narcissistic, Antisocial and Passive-Aggressive styles were co-loaded on one broad antisocial domain [43]. Although the association between the Intelligent trait and the Antisocial style was weaker than in other styles, in Chinese culture, the antisocial personality trait covers one part of fierce and malicious, resembling the Machiavellian attitude [44]. The latter was correlated with the intelligence in western culture [45].

Fourthly, the Obsessive-Compulsive style was strongly predicted by the Conscientious trait in patients. It has been shown that the conscientiousness predicted workaholic characteristics [46], and workaholics scored higher on obsessive-compulsive personality disorder than healthy participants did [47]. Moreover, the obsessive-compulsive personality disorder presents a maladaptive variant of normal-range conscientiousness [48, 49].

Lastly, the Antisocial (-) style was strongly predicted by the Agreeable trait together with Unsocial in patients. A recent study has shown that the Agreeableness in the Five-Factor Nonverbal Personality Questionnaire [50] also strongly predicted the Antisocial (-) style [51]. Meantime, the Agreeable trait showed its capability of predicting the Antisocial style in our healthy participants. These associations again support that the agreeableness and antisocial traits are regarded as two opposite poles of a personality domain in both clinical and normal populations [52, 53].

One has to acknowledge that the current study is a preliminary design and suffers at least three limitations. Firstly, subsample sizes of individual personality disorders were relatively small and not equally distributed in number and in age, a larger recruitment of patients would make the current relationship clearer. Secondly, the internal reliability of the PERM Schizoid style was lower as usual, which might also influence the current results. Thirdly, we did not include a group of non-Chinese people, which would be a nice control for the current results. Fourthly, the participants were young people aged 18-26 years, and the results may not be generalizable to other age populations. Nevertheless, our study has demonstrated that all five CADP traits were correlated with almost all 11 PERM styles, and the associations in patients were more specific and clearer. Therefore our study indicates a validity of CADP for predicting personality disorder types, and warrants its application as an aid to diagnose personality disorders in China.

## Conclusions

Our preliminary study has demonstrated that, in personality disorder patients, all five CADP traits were specifically associated with almost all 11 PERM styles, and the associations in patients were specific and clear. Therefore, the validity of CADP for predicting personality disorder types indicates that CADP might be used as an aid to diagnose personality disorders in China.

## Abbreviations

CADP: Chinese Adjective Descriptors of Personality
PERM: Parker personality measure
PVP: Plutchik-van Praag depression inventory
ZKPQ: Zuckerman-Kuhlman personality questionnaire.
